# By-Catch of Grey Seals (*Halichoerus grypus*) in Baltic Fisheries—A Bayesian Analysis of Interview Survey

**DOI:** 10.1371/journal.pone.0113836

**Published:** 2014-11-25

**Authors:** Jarno Vanhatalo, Markus Vetemaa, Annika Herrero, Teija Aho, Raisa Tiilikainen

**Affiliations:** 1 Department of Environmental Sciences, University of Helsinki, Helsinki, Finland; 2 Estonian Marine Institute, University of Tartu, Tartu, Estonia; 3 Finnish Game and Fisheries Research Institute, Helsinki, Finland; 4 Department of Aquatic Resources, Swedish University of Agricultural Sciences, Öregrund, Sweden; 5 Metsähallitus, Savonlinna, Finland; Texas A&M University-Corpus Christi, United States of America

## Abstract

Baltic seals are recovering after a population decline. The increasing seal stocks cause notable damage to fisheries in the Baltic Sea, with an unknown number of seals drowning in fishing gear every year. Thus, sustainable seal management requires updated knowledge of the by-catch of seals—the number of specimens that die in fishing gear. We analyse the by-catch of grey seals (*Halichoerus grypus*) in Finland, Sweden, and Estonia in 2012. We collect data with interviews (35 in Finland, 54 in Sweden, and 72 in Estonia) and analyse them with a hierarchical Bayesian model. The model accounts for variability in seal abundance, seal mortality and fishing effort in different sub-areas of the Baltic Sea and allows us to predict the by-catch in areas where interview data was not available. We provide a detailed description of the survey design and interview methods, and discuss different factors affecting fishermen's motivation to report by-catch and how this may affect the results. Our analysis shows that the total yearly by-catch by trap and gill nets in Finland, Sweden and Estonia is, with 90% probability, more than 1240 but less than 2860; and the posterior median and mean of the total by-catch are 1550 and 1880 seals, respectively. Trap nets make about 88% of the total by-catch. However, results also indicate that in one of the sub-areas of this study, fishermen may have underreported their by-catch. Taking the possible underreporting into account the posterior mean of the total by-catch is between 2180 and 2380. The by-catch in our study area is likely to represent at least 90% of the total yearly grey seal by-catch in the Baltic Sea.

## Introduction

Baltic seals are recovering after a population decline in the late 20^th^ century. However, they face a changed ecosystem both in terms of human-induced mortality (hunting and by-catch in fishing gear) and in availability of food resources. On the other hand, the increasing seal stocks cause notable financial loss to coastal fisheries [1], [2], especially in the northern Baltic Sea. Lack of updated information on different aspects of the interactions between seal and fish stocks and coastal fishery aggravates seal-fishery co-existence and the accomplishment of a favourable conservation status. As the conflict with coastal fishery has arisen [3], licensed hunting has been reintroduced in Finland and Sweden, and a stronger regulation of the seal population has been called for by fishermen. However, the by-catch of seals – the number of specimens that die in fishing gear is unknown, complicating the assessment of a sustainable hunting quota and the conservation actions needed.

The only earlier data sources about seal by-catch in the Baltic Sea are interviews of fishermen by Lunneryd et al. [4]. The authors extrapolated that in 2001 the number (and 95% confidence interval) of by-caught seals in Swedish waters was 462 (360–575) grey seals, 52 (34–70) ringed seals and 461 (333–506) harbour seals. However, there is no estimate for the total by-catch of seals in the Baltic Sea. Moreover, in parallel to the growing number of Baltic grey seals, the number of animals drowning in fishing gears might also have increased. Hence, sustainable management both on national and international levels requires broader and updated knowledge on the by-catch of seals in the Baltic.

For large-scale fisheries with high economic values by-catch data is often achieved by independent observers recording by-catch on-board, which is later extrapolated to the whole fishery. Obtaining information from small-scale fisheries like the Baltic coastal one is more problematic. The small economic value of fisheries combined with the low frequency of by-catch makes the usage of observers practically impossible. Even if few by-catch events could be detected, extrapolation of such episodic data would have low statistical reliability. Due to these constraints, by-catch data from small-scale fisheries is usually based on interviews [5], [6], [7], which is the chosen data collection method in this study as well. This leads, however, to greater uncertainty and lower credibility in data compared to data collected by observers and thus requires a rigorous analysis.

In this work, we analysed the by-catch of grey seals (*Halichoerus grypus*) in coastal trap nets and gill nets used by professional coastal fisheries in Finland, Sweden, and Estonia in 2012. According to our study, these gears are responsible for most of the by-catch in the Baltic Sea. Still, trawls cause some sporadic mortality too. We collected data with interviews (35 in Finland, 54 in Sweden and 72 in Estonia) and analysed them with a hierarchical Bayesian model. The model accounts for variability in seal abundance, seal mortality and fishing effort in different sub-areas of the Baltic Sea and allows us to predict the by-catch in areas where interview data was not available. We provide a detailed description of the survey design and interview methods, and discuss different factors affecting fishermen's motivation to report by-catch and how this may affect the results.

## Methods

### Study area and gears considered

**Figure 1:**
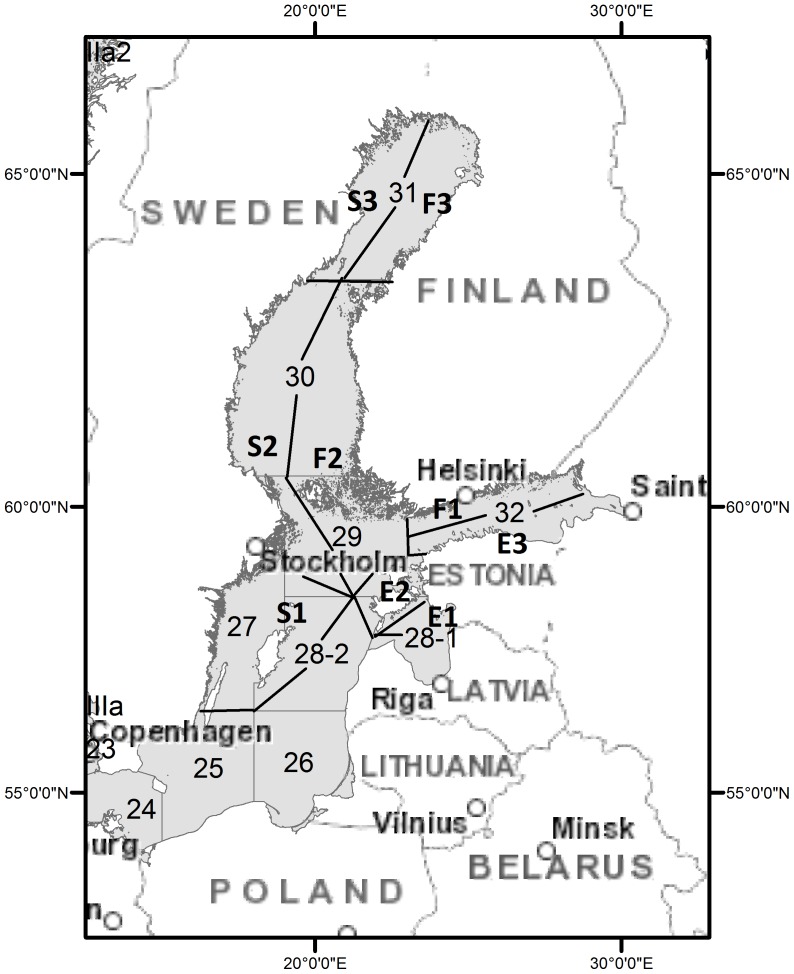
The study area and its division into 9 subareas.

The choice of the specific types of fishing gear to be included in this study was based on the interviews; earlier experience of seal and fisheries researchers in Finland, Estonia and Sweden, and annual notifications of claims for seal-induced harm by fishermen. We excluded gear types for which no by-catch or only sporadic by-catch have been reported. Our study area covers the central and northern Baltic (approximately the ICES statistical squares 27–30 and 32), which is the main distribution area of Baltic grey seals. (Fig. 1). Since both the fishing methods, as well as the abundance of seals, vary across the Baltic Sea, we aggregated the data into 9 coastal sub-areas (Fig. 1, Table 1). The division was done so that within each sub-area the coastal fishing methods and environmental conditions are homogenous enough to assume constant (average) by-catch mortality and seal abundance. This was done based on the fisheries statistics and ecosystem-based division of the Baltic Sea in the literature (see e.g., [8]).

**Table 1:** The survey and effort data and the estimated proportion of seals from the total population in each sub-area.

	Trap nets			Gill nets				proportion of seals		
sub-area	by-catch in sample	effort in sample (gear-days)	total effort (gear-days)	by-catch in sample	effort in sample (km-days)	total effort (km-days)	number of interviews	expected (%)	spring (%)	fall (%)
E1	104	2458	11772				34	6.25	7.5	5
E2	59	1542	8722				25	6.25	7.5	5
E3	10	1096	3135				13	1.75	0.5	3
F1	45	23533	36636				25	1.75	0.5	3
F2	5	3823	58863				10	30	35	25
F3	0	0	5057				0	2	0	4
S1	0	0	8515	20	1402	7904	13	22.5	25	20
S2	61	2450	2563	35	1540	1611	41	16.5	15	18
S3	0	0	4806				0	2	0	4
total	284	34902	140069	55	2942	9515	161	89	91	87

Empty gill net cells represent areas where we assume gill nets do not contribute to by-catch.

### Data

The data was obtained through interviews of fishermen in 2012–2013 and from databases of national authorities. We also interviewed 5 fisheries and seal experts (2 seal monitoring researchers, 1 fisheries spokesman, and 2 conservationists) in order to elicit prior distributions for model parameters. In order to diminish scepticism and distrust in fishermen, which could lead to underreporting, we directly contacted fishermen who had the highest catches, who were classified by national standards as professionals (e.g. over 30% of annual income in Finland) and who had frequent contacts with researchers. In Finland, we also advertised our interviews among all fishermen so that anyone willing to be interviewed would be included into the panel. This resulted in 35 fishermen interviewed in Finland, 54 in Sweden and 72 in Estonia. In order to assist honest reporting, most of the interviews were conducted face-to-face, and anonymity was granted to interviewees. In Sweden, approximately 80% of the interviews were made face-to-face and 20% by sending the questionnaire by mail. In Estonia, 50% of the interviews were made face-to-face and 50% by telephone. In Finland, 33 interviews were made face-to-face and one by telephone. Interviews were recorded by filling in the questionnaire form, and they were conducted by 4 people; one in Finland, one in Sweden, and two in Estonia. Three of the interviewers were female and one male. The set of questions and other details concerning interviews were mutually agreed before the interviews so that the content of all interviews was the same.

The personal information gathered from the participants included their name, age, address and fishing region. This information was used to link the fishermen with national fisheries information systems from where the fishing effort in the interviewed sample was calculated. However, for the purposes of this study the individual answers and the fishing efforts were aggregated so that individual participants could not be identified. Data on fishing effort was obtained from the respective national authorities: the Finnish Game and Fisheries Research Institute, the Swedish Agency for Marine and Water Management, and the Estonian Ministry of Agriculture. The fishing effort was calculated for trap nets in gear-days and gill nets in km-days.

The general questions posed to fishermen were: how many years they had fished professionally, what fishing methods (e.g. gear) they used and which species they fished, their description of harm caused by seals to fisheries and fishing gear, what time of the year seals caused problems, had seal induced harm increased and had fishermen changed their fishing method because of seals. The specific questions on by-catch were: did fishermen catch seals as by-catch, with which gears and how often did they catch seals, how many seals did they catch in 2012, what time of the year did most by-catch occur, of what species (grey or ringed seal) the by-caught seals were and had by-catch increased during recent years. After this, fishermen were asked about seal harm mitigation: had harm caused by seals in their fishing region been mitigated by changing fishing gear or by hunting, did fishermen use bars in front of their traps, repellents or other means to protect their gear and had these methods been successful?

The ages of interviewed fishermen ranged from 23 to 77 years. All provided their verbal informed consent to participate in this study and knew that their responses would be used as a part of potentially published research. The informed consent was implicitly recorded by the fact that fishermen participated in the interviews since those who did not provide consent were not interviewed. Written consent was not obtained since it was not required for this type of study by the national rules concerning ethics in research. Based on the rules of the Finnish Advisory Board on Research Integrity, this consent procedure and the study design are exempt from prior ethical approval by an ethics committee. The aggregated data is summarised in Table 1. We did not have permission to publish the raw data but Table 1 lists all data necessary to redo the analysis.

We excluded sub-areas F3 and S3 from the survey for practical reasons. The funding and time budget of this research did not allow the inclusion of these sub-areas into the survey. For the same reason we excluded Russia, Latvia and southern parts of the Baltic Sea. However, on the basis of our results, we discuss their share of by-catch as well.

### Analysis

We applied Bayesian methods to analyse the data. The benefits from using Bayesian analysis are that we can explicitly and transparently state our assumptions and uncertainties about the phenomenon and data in probabilistic form. The results of an analysis are posterior distributions which provide estimates for the by-catch and model parameters and describe the uncertainty in them.

We have divided the total study area into sub-areas (Figure 1) so that they correspond to the different kind of gears and use of those gears in the Baltic Sea. Hence, it is reasonable to model the by-catch within each sub-area separately. We modelled the average mortality rates of a seal per unit effort (catchability) with a trap or a gill net as exchangeable between sub-areas. We also assumed that by-catch mortality is additive to other mortality sources, and that, given the catchability, the probability of a seal surviving a unit effort of fishing is independent of the fishing effort it has already survived. Then, the fishing effort a seal survives (“lifetime” of a seal) will be exponentially distributed, and the probability for a seal in sub-area *a* to die via by-catch is 
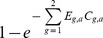
, where *E_g,a_* is the total effort and *C_g,a_* is the catchability of a gear *g* in that sub-area. The catchability accounts for the process of a seal encountering a gear and becoming entangled in it. Since the number of seals in a sub-area may vary within a year we parameterize the model with an average (effective) number of seals in sub-areas. Then, the number of by-caught and survived seals in a sub-area will be 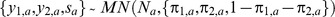
where *N_a_* represents the average number of seals in sub-area *a* that survive other reasons for mortality, *y_g,a_* is the number of seals that died in gear *g*, *s_a_* is the number of surviving seals, and 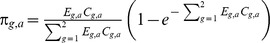 is the probability of dying in gear *g* in one year.

We gave a hierarchical prior for the catch-abilities, *p(C_g,a_*), and number of seals, *p(N_a_)*, and calculated their posterior distribution using the Bayes rule
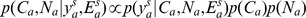
where 
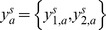
 and 
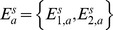
 are the by-catch and effort in the survey respectively, and 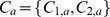 collects the catch-abilities of both gears. After this we calculated the posterior predictive distribution of the by-catch by fishermen that were not interviewed
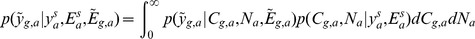
where 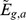 is the gear and sub-area specific fishing effort of fishermen that were not interviewed. The total by-catch is then the sum of the by-catch in the survey and the predicted by-catch by the remaining fishermen, 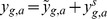.

Baltic grey seals are counted annually during their peak moulting time in early summer. The population is increasing and in 2012 there were 28255 counted individuals [9]. Hiby et al. [10] estimated that the proportion of counted individuals is 70%–85% of the total population. Hence, it is reasonable to assume that the number of counted seals is a conservative minimum and 28255/0.7≈40000, an optimistic maximum estimate of 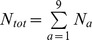
– the total number of seals that survived other mortalities than by-catch in 2012. We encoded these assumptions by a log-Gaussian prior
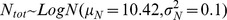
where 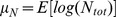
and 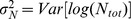. This gives 95% probability for values less than 40000 and 95% probability for values more than 28000, with mean 34000≈28255/0.85.

The division of the total seal population between sub-areas was based on annual counts and expert assessment as follows. The number of seals in each sub-area during the survey was calculated from the survey counts, with two exceptions. The survey counts reported the total number in E1 and E2, which was evenly divided between them. In survey counts the area south from Stockholm was combined with S1 and, thus, we estimated that 30% of the counted seals in that area belonged factually to sub-area S2 of our study. We interviewed a researcher responsible for the survey counts in Finland (Markus Ahola, Finnish Game and Fisheries Research Institute) in order to estimate the change from the number of individuals during surveys to that in fall. During surveys seals are aggregated in archipelago areas, which are good for moulting, whereas in fall seals distribute throughout the northern Baltic Sea to forage. The redistribution was done roughly in proportion of the area not suitable for moulting in each sub-area. These estimates were transferred to proportions of seals from the total population by dividing them by the total number of counted animals. The larger (smaller) from survey and fall estimates was used as maximum (minimum) estimate of the number of seals in each sub-area (Table 1). The uncertainty about the sub-area proportions were encoded into the model using Dirichlet distribution as follows.

The expected proportion of seals in each sub-area was the mean of the minimum and maximum estimates (Table 1). The scale parameter of the Dirichlet distribution was chosen so that approximately 90% of the prior probability mass was between the minimum and maximum estimates in the case of sub-area with the highest proportion (sub-area F2). Since, due to the properties of Dirichlet distribution, the variance relative to mean is greater for smaller proportions only about 60% of prior probability mass is within min/max values in the case of sub-areas with an expected proportion less than 10%. Given the total number of seals and the proportion of seals in sub-areas, the average number of seals in them was assumed to follow multinomial distribution. This led to Dirichlet Multinomial prior for the average number of seals in sub-areas


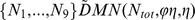
, where 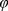 denotes the vector of prior expectation of proportions (Table 1) and the scale 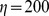 governs the uncertainty about the expected value.

The catchability of a gear depends on many things, such as the specific type of gear and the fish species it targets. However, despite the evident variability in sub-area specific catchabilities they are still related. This was modelled by giving a hierarchical prior [11] for the scaled catchabilities. For computational reasons we implemented the model by down-scaling the effort by 10^−4^ but all the results are reported in the original scale. The prior for the catchabilities was 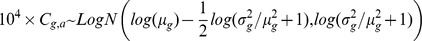


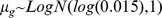


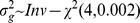
where 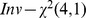
is the inverse Chi squared distribution with 4 degrees of freedom and scale 1 [11]. Here the area specific catchabilities depend on population mean 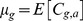
and variance 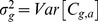 which define the prior expected catchability of all gears *g* used in the Baltic Sea and across sub-areas variation around it. The hyper-priors for the population parameters were set as follows. Based on expert (2 seal monitoring researchers, 1 fisheries spokesman, and 2 conservationists) assessment, the total by-catch in the Baltic Sea was of order few hundreds at minimum to few thousands at maximum. Their estimates ranged from 500 to 2000 with mean 1200 by-caught grey seals in total. A catchability of 1.5×10^−6^ or 8×10^−6^ (in the original scale) for both trap nets and nets in each sub-area, would lead to approximately 1200 and 5500 by-caught seals, respectively. The former represents the mean estimate of experts and the latter can be used as a pessimistic upper limit of the mean catchability since such a high number of by-caught seals is unlikely when compared to the total population size and the fact that the population is increasing. Thus, the prior was set so that with 95% probability 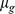 is (in the original scale) below 10^−5^ and its prior median is 1.5×10^−6^. We assumed that the coefficient of variation in the catchabilities is likely over one but unlikely to be much more than ten. Hence the prior for 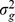 was set so that with 95% prior probability the coefficient of variation was more than 0.6 and less than 19 with prior median 3.4. The resulting marginal prior for 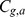 has 95% of its probability mass over 0.3×10^−6^ and under 8×10^−6^.

The posterior inference was then conducted for the parameters and population parameters. The hierarchical prior allowed us to predict, based on the posterior of the population parameters, the catchability in sub-areas F3 and S3, where interviews were not made. Since we had effort data for these sub-areas we could calculate the posterior predictive distribution of the by-catch there as well. We approximated the posterior distributions of model parameters with Markov chain Monte Carlo using the Metropolis-Hastings algorithm [12].

## Results

### Gears causing by-catch

In Estonia, only two types of trap net cause considerable by-catch: open-sea fykes with mouth size over 3 m, and coastal fykes with mouth size 1–3 m. These were included in the analysis in sub-areas E1-E3. Some fishermen interviewed in Estonia also described the rare drowning of pups in fyke nets with a smaller mouth size, and in salmon gill nets, but since there were no such reports for year 2012, these were excluded. In Finland practically all by-catch is caused by push-up trap-nets with mouth openings larger than 3 m. Hence, in sub-areas F1-F3 we considered only this type of gear. In Sweden, the by-catch is mainly caused by similar push up trap nets to those in Finland, but in sub-areas S1 and S2 also by several types (different mesh sizes) of gill nets. In sub-area S1 (Fig. 1), the gill nets are mainly used for fishing cod and flatfish whereas in sub-area S2 they target mainly whitefish and herring. Trap nets were included in the analysis in sub-areas S1-S3 and gill nets in sub-areas S1 and S2. Few fishermen in Sweden also reported sporadic by-catch in trawls, but due to the very low share of this type of by-catch, trawls were excluded from the analysis. Recreational fishing causes negligible by-catch at most since recreational fishermen do not use trap nets. Moreover, in sub-areas S1 and S2 their nets are weaker than those used by professional fishermen and, hence, seals do not drown in them to the same extent.

### Number of by-caught seals

**Figure 2:**
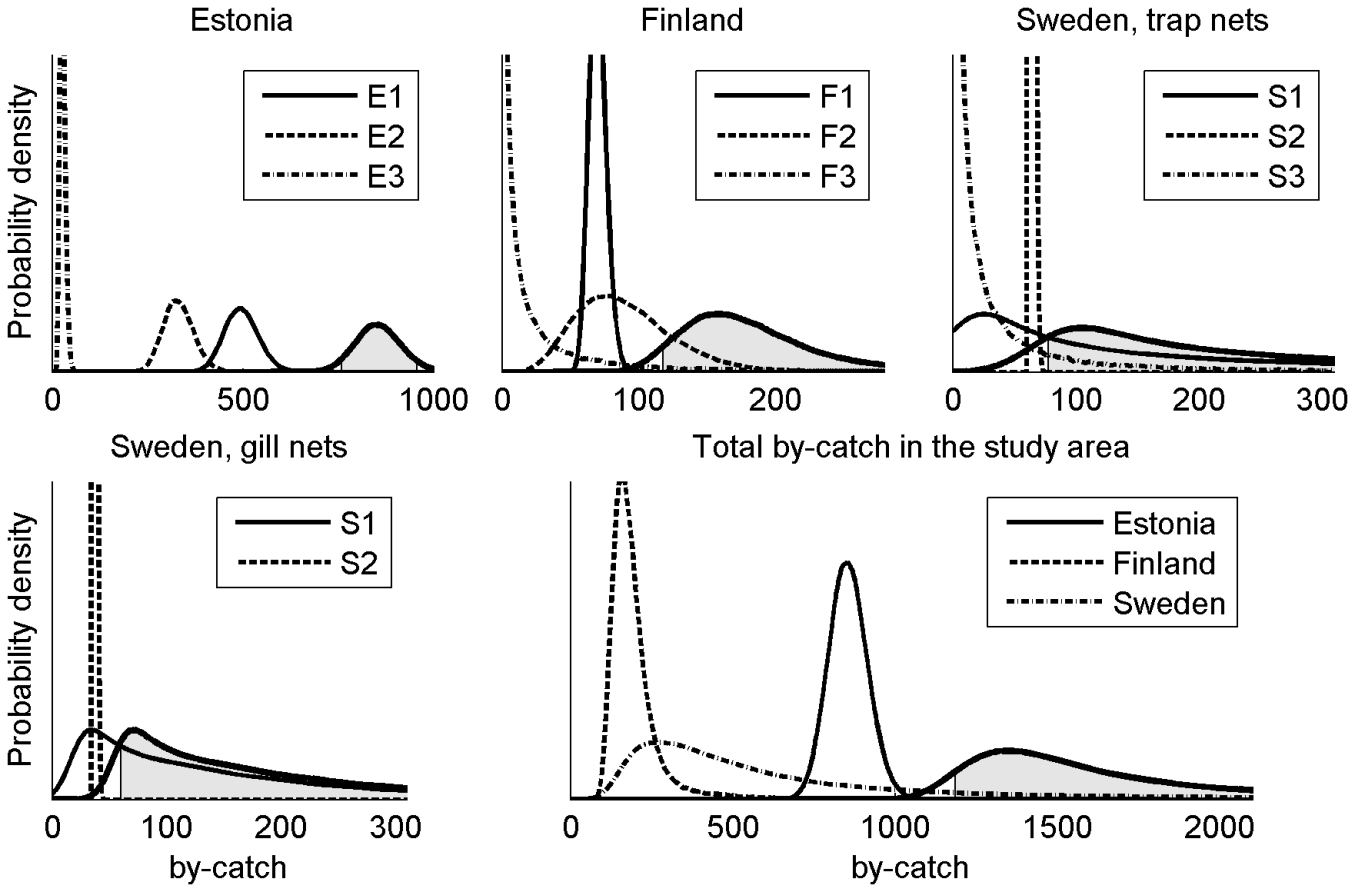
The posterior distributions of the by-catch. In the country specific panels, the lines without fill represent by-catches in sub-areas and the line with grey fill represents the total by-catch in a country. In the total by-catch panel, the lines without fill represent the country-specific by-catches (filled lines in country panels) and the line with fill represents the total by-catch in the Baltic Sea. The grey shaded area is the central 90% credible interval.

The total by-catch by trap and gill net fisheries in our study area was, with 90% probability, more than 1240 but less than 2860, and the posterior median and mean were 1550 and 1880 seals respectively. The posterior distribution was highly right skewed (Fig. 2) and the 80% quantile of the total by-catch was 2130. With 90% probability, the total by-catch with trap nets was more than 1100 but less than 2600, and that of gill nets more than 70 but less than 410. The posterior mean of the proportion of by-catch with trap nets from the total by-catch was 88%.

With 90% probability the by-catch in Estonia was more than 780 but less than 930, and in Finland more than 130 but less than 270. In Sweden, the posterior distribution of the by-catch was highly right skewed and there the by-catch was with 90% probability over 210 and under 1790. However, the 80% quantile of the posterior of the by-catch in Sweden was 1060.

**Figure 3:**
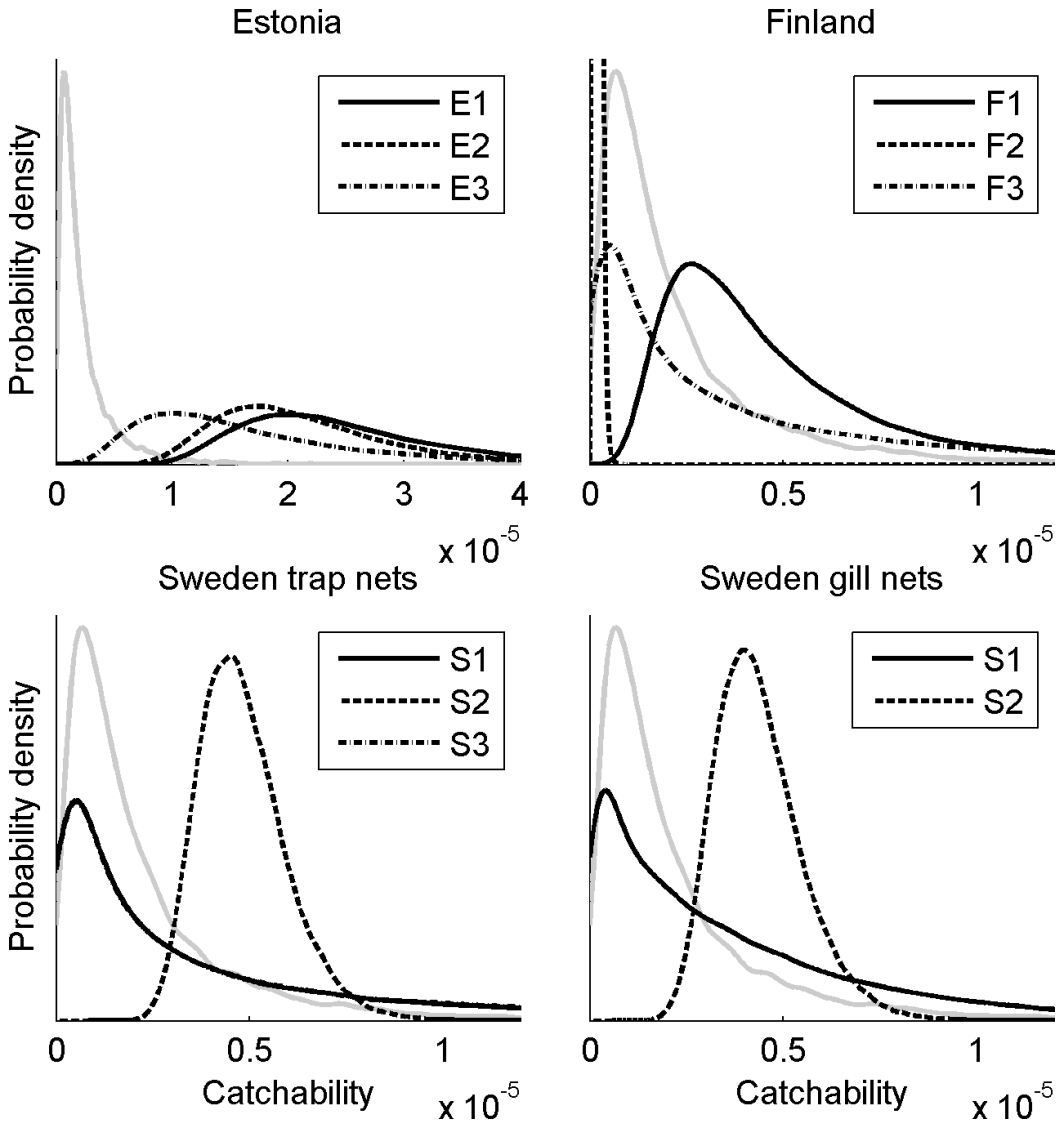
The posterior (black lines) and the marginal prior (grey line) distributions of catchabilities 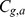.

**Figure 4:**
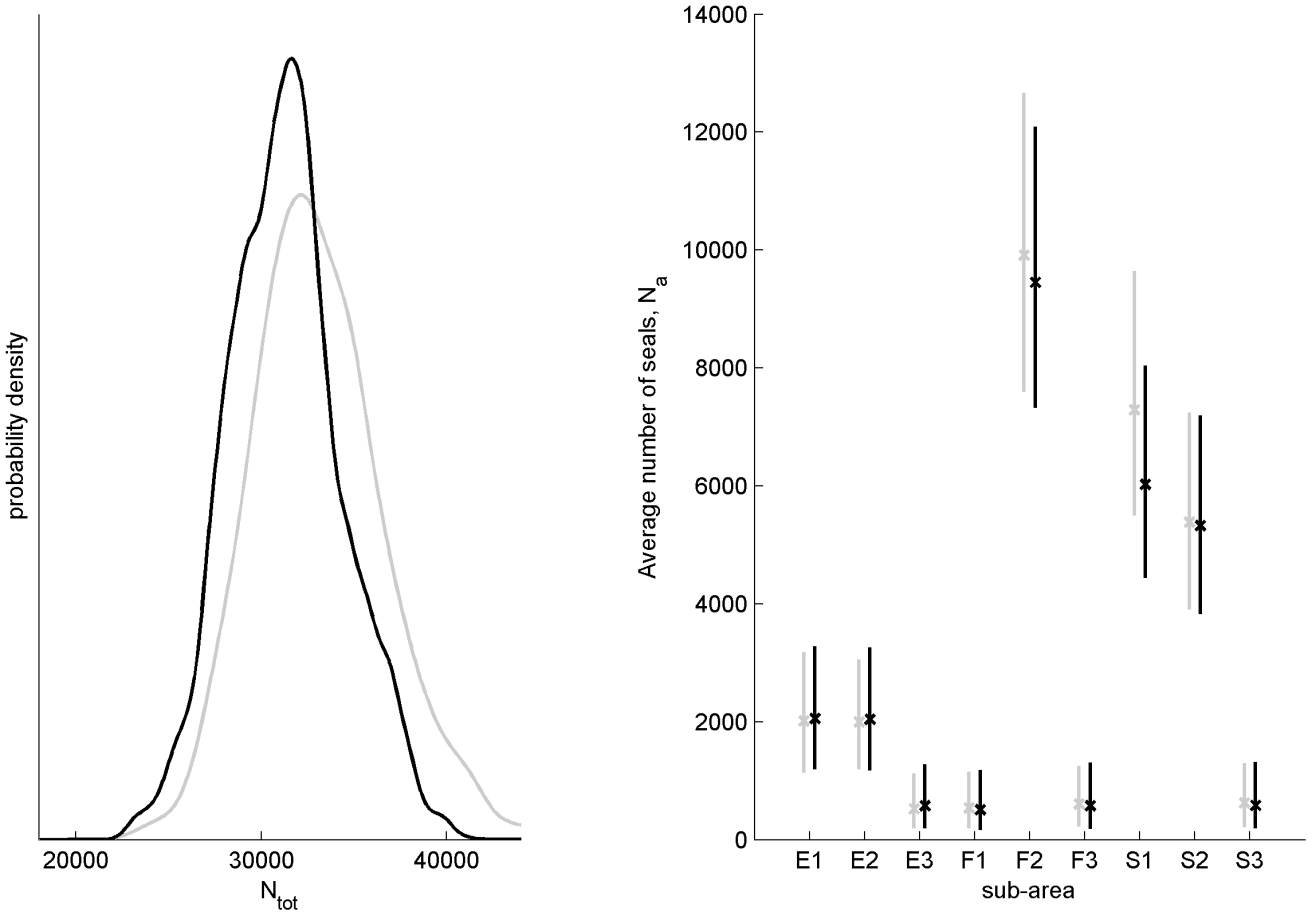
On the left, the posterior (black) and prior (grey) distribution of the number of seals that have survived other mortality sources than by-catch. On the right, the posterior (black) and prior (grey) median (cross) and central 90% credible interval of the average number of seals in sub-areas.

The main reason for the heavily right skewed posterior of the by-catch in Sweden was the uncertainty in the posterior distributions of catchabilities of gill nets and trap nets in S1 (Fig 3.), where the average seal abundance was second highest (Fig. 4). The posterior of the gill net catchability in S1 was concentrated in smaller values than in S2. However, since the gill net effort in the sample relative to the total effort in S1 was small compared to that in S2, the posterior of gill net cacthability in S1 was more uncertain leading to heavy right tail. This caused the posterior of the gill net by-catch in S1 to have heavy right tail as well. Similarly, since there were no trap nets included in the survey from S1, there was large uncertainty in posterior of their catchability there. This was reflected by heavy right tail which caused heavy right tail in the posterior of the trap net by-catch in S1.

The posterior of the total number of seals that survived other mortality sources, 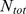, is concentrated on smaller values than its prior. The posterior distributions of population sizes in sub-areas followed closely their prior everywhere else except in S1 (Fig. 4). In S1, the population size has decreased, compared to the prior.

The catch-abilities of trap nets varied from the order of magnitude 10^−7^ in Finland to 10^−5^ in Estonia. However, in all other surveyed areas except F2 the catchability was with 95% probability greater than 10^−6^. The catchability in sub-area F2 was with 95% under 3×10^−7^ and, thus, this sub-area differed substantially from other sub-areas. The posterior predictive distribution of trap net catchability in sub-areas F3, S1 and S3, from where we did not have interview information on them, was concentrated near zero with median 3×10^−6^ and a heavy right tail (Fig. 3). The high variance in these distributions was due to high variability in catch-abilities between sub-areas with observations. The uncertainty in catchability was the smallest in S2, where almost all fishermen were interviewed.

We also conducted a sensitivity test for the priors over population size and its division into sub-areas as well as catchability. In general, the estimates of by-catch in sub-areas where surveys were done were not sensitive to the priors. However, when catchability priors were made wider or the expected proportion of population in sub-areas S1 or S2 were altered more than 30% from their values in Table 1, the posterior distributions of catchabilities in sub-areas where surveys were not made became more uncertain leading to heavier tailed posterior for the total by-catch. The population size affects the coefficient of variation of the observation model so that smaller population size leads to higher coefficient of variation and thus larger posterior uncertainty about the by-catch estimate. This increase was not significant within about 50% increase or decrease of the prior mean of the total population size.

## Discussion

Evaluating by-catch based on interviews is a challenging task since fishermen have a clear motivation to hide information. While high by-catch numbers may be used by administrations as a reason to pose additional restrictions such as time or area closures for fisheries, nothing “good” to a fisherman can emerge from his outspokenness. As a result, the numbers obtained through interviews often reflect minimum by-catch rates rather than give exact data about the real values. Such a conclusion was recently stressed e.g. by Dmitrieva et al. [13], who analysed by-catch of Caspian ringed seals, and came to the conclusion that the obtained yearly by-catch estimate of 1225 seals (1,2% of the population size) was probably several times or even an order of magnitude smaller than the real figure.

The motivation for under-reporting was also the main risk in the current study. However, in Estonia for example, more active fishermen with higher catches were selected for the panel. As these fishermen have regular contacts with scientists who posed the questions, we also knew most of them well and could conclude that distrust was not a serious issue in Estonia. Moreover, as most fishermen interviewed had also been selected for panels on similar issues before, they knew that questionnaires had not been used as a reason to pose fishing restrictions. Finally, in most cases the data submitted by fishermen was in good concord with the expert opinion of researchers having long experience in the topic. Generally, this also applies to the situation in Sweden.

In Finland, fishermen's motivation to report by-catch has generally been, and was also in the current interviews, very low. There are several reasons for this. Firstly, fishermen feel that they have not gained anything by supporting earlier similar studies. Secondly, they believe new fishing restrictions are often made and that the general attitude in society towards fishermen is negative. As an example, coincidently the interviews were made during an abundant salmon year, when the Finnish World Wildlife Fund initiated a campaign against eating wild salmon and whitefish, which rapidly caused the biggest food markets to stand back from buying wild fish. Moreover, discussions on whether mesh size in pikeperch nets should be enlarged took place at the same time.

In order to mitigate the sceptical or even hostile attitude of fishermen, in Finland all the interviews but one were made face-to-face, which encouraged more relaxed talk compared to telephone interviews. Moreover, the questions regarding by-catch were posed at the end of the interviews. Still, while the by-catch numbers obtained from Sweden and Estonia appear trustworthy, the Finnish figures may be underestimated. Finnish fishermen themselves explained low figures by the many technical innovations undertaken during recent years. Indeed, in Sweden many fishermen also highlighted the fact that the by-catch has decreased along with improvements in gear technology.

The discrepancy between Sweden and Finland needs more attention in the future. In our opinion it is unlikely that the catchability of trap nets in Sweden and in sub-area F1 could be over 10 times greater than in sub-area F2 since the gears and their use in sub-area F2 do not differ much from that in Sweden and in sub-area F1. The low catchability in sub-area F2 could also be explained by inflated population size estimate there. However, if catchability in sub-area F2 was of the same magnitude as in Sweden and in sub-area F1, the number of seals in sub-area F2 should be less than 10% from its current estimate, which we think is very unlikely. If the catchability in sub-area F2 was of the same order as in Sweden and in sub-area F1, the by-catch in Finland would be 300–500 individuals higher than reported in our study. Then, the posterior mean of the total by-catch in our study area would be between 2180 and 2380.

Analysing by-catch depends heavily on the assumptions made about data collection and the phenomena related to it, such as fishing gear and behaviour of fishermen. Bayesian probability theory provides tools with which these assumptions can be explicitly and transparently coded into a mathematical model, and merged with the information contained by data. The posterior distributions summarise the analyst's understanding of the phenomenon after merging his/her prior information with new data. Moreover, this approach allows us realistically to predict the by-catch in sub-areas where we could not conduct interviews but from where we have effort data.

We were not able to extrapolate the by-catch to the remaining coastal areas in the Baltic Sea due to the lack of fishing effort data. However, in Russia fishery with large traps is not widespread. Due to the open coastline, which exposes traps to occasional storms, the same applies to Latvia and Lithuania. Moreover, an average of only 9–13% of the seal population is outside our study area (Table 1); therefore, it is likely that the by-catch covered in our study represents at least 90% of the total fisheries-induced mortality of grey seals (trawls included) in the Baltic Sea. Thus, if possible under-reporting in Finland is also taken into account; the total yearly by-catch of the Baltic grey seals seems to be in the order of 2000 animals or more.

Pups and young seals make up most of the by-catch registered during the present study (Kauhala Kaarina unpublished data). High mortality of young seals is in good accordance with data presented by Bjørge et al. [14], who concluded that in Norway, seals are most vulnerable to incidental mortality in fishing gear during the first three months after birth, and that high incidental mortality prevails during the first 8–10 months. The low share of older animals in by-catch is most likely the reason why the Baltic grey seal population has been growing despite the high by-catch number – pups also have an otherwise much lower survival rate than adults. Moreover, by-caught animals are not a random sample of the population. In general, they are in inferior condition and probably face a higher death rate otherwise as well (Kaarina Kauhala unpublished data).

Lunneryd et al. [4] estimated that 462 (360–575) grey seals were by-caught with all gears (including trawls) in Sweden in 2001. The counted grey seal population in 2001 was 10300, but according to the expert interview the counting efficiency was less than in 2012. Thus, the population size of Baltic grey seals is now at most three times larger than in 2001. Based on this, the by-catch mortality seems to have decreased since 2001 in Sweden, which is in accordance with the fishermen's opinion and supposed to be largely due to the development made in fishing gears and methods.

In all three study countries fishermen are obliged to note mammal by-catch events in their statistics. However, the figures presented in the current study clearly differ from the official statistics – e.g. in Estonia officially registered by-catch is zero in most years. Hence, the data gathered routinely by the administrations based on how much fishermen volunteer, is incomplete and cannot be used in management.

What is the reason behind these weak statistics? The seal population has increased almost ten-fold compared to the numbers counted in the late 1970s [15]. Since the majority of fishermen are older than 50, most of them have witnessed how seal damage has grown from practically zero to that which causes substantial financial harm; for example, in 1995 seal damage was already regarded as a cause of major economic loss to the Finnish fishery [3]. Several attempts have been made to construct special gears, with the aim of decreasing by-catch as well as diminishing losses through seal attacks [16], [17]. Still, since modified traps can protect only the catch already in the gear, seals have learned to hunt for fish in the mouths of the traps. Even if there are some governmental aid systems to compensate part of the damage, the financial losses borne solely by fishermen themselves have been steadily increasing. The great majority of fishermen in our study complained for damaged gears and lost catch. Moreover, many of them believe that revealed high by-catch numbers are likely to be used as a cause to pose additional fishing restrictions, which will decrease profitability even more. If we add this attitude to the deteriorating economic situation in coastal fisheries, it becomes clear why the willingness of fishermen to co-operate with scientists and administrations is low.

The present study gathered rather reliable data on by-catch, but how could we improve the annual statistics in the future? One option could be to increase enforcement. However, given that fishermen work alone on the sea, this is not realistic. The other option is to seek fishermen's higher voluntary compliance. To achieve this, solidarity should be shown and a fair share of the negative impact to fisheries of the “environmental good” of increasing seal numbers should be borne by the rest of the society, especially, since the increasing seal population has been set as a management objective by the Baltic Sea countries [18]. It can be concluded that while this is not met, and fishermen are left alone with growing problems, it is not realistic to expect that they will start to provide better data on by-catch.

## References

[1] Königson S, Lunneryd SG, Stridh H, Sundqvist F (2007) Grey seal predation in cod gillnet fisheries in the Central Baltic Sea. Journal of Northwest Atlantic Fishery Science 42:41–47 10.2960/J.v42.m654

[2] Königson S, Fjälling A, Berglind M, Lunneryd SG (2013) Male gray seals specialize in raiding salmon traps. Fisheries Research 148:117–123 10.1016/j.fishres.2013.07.014

[3] Varjopuro R (2011) Co-existence of seals and fisheries? Adaptation of a coastal fishery for recovery of the Baltic grey seal. Marine Policy 35:450–456 10.1016/j.marpol.2010.10.023

[4] Lunneryd SG, Hemmingsson M, Tärnlund S, Fjälling A (2005) A voluntary logbook scheme as a method of monitoring the by-catch of seals in Swedish coastal fisheries. In *ICES CM* (X:04). International Council for the Exploration of the Sea, Köpenhagen, Denmark.

[5] Sipilä T (2003) Conservation biology of Saimaa ringed seal (*Phoca hispida saimensis*) with reference to other European seal populations. PhD. thesis, University of Helsinki.

[6] Karamanlidis AA, Androukaki E, Adamantopoulou S, Chatzispyrou A, Johnson WM, et al (2008) Assessing accidental entanglement as a threat to the Mediterranean monk seal *Monachus monachus* . Endangered Species Research 5:205–213 10.3354/esr00092

[7] Moore JE, Cox TM, Lewison RL, Read AJ, Bjorkland R, et al (2010) An interview-based approach to assess marine mammal and sea turtle captures in artisanal fisheries. Biological Conservation 143:795–805 10.1016/j.biocon.2009.12.023

[8] Ojaveer E, Kalejs M (2008) On ecosystem-based regions in the Baltic Sea. Journal of Marine Systems 74(1–2):672–685 10.1016/j.jmarsys.2008.07.001

[9] Ahola M, Merihylkeet V (2012) In: Wikman, M. (ed.), Monitoring game abundance in Finland in 2012. Riista- ja kalatalous – Tutkimuksia ja selvityksiä. in press.

[10] Hiby L, Lundberg T, Karlsson O, Watkins J, Jüssi M, et al (2007) Estimates of the size of the Baltic grey seal population based on photo-identification data. NAMMCO scientific publications 6:163–175.

[11] Gelman A, Carlin JB, Stern HS, Dunson DB, Vehtari A, et al.. (2013) Bayesian Data Analysis Third Edition. Chapman&Hall/CRC.

[12] Gilks ER, Richardson S, Spiegelhalter DJ (1996) Markov chain Monte Carlo in practice. Chapman & Hall/CRC.

[13] Dmitrieva L, Kondakov AA, Oleynikov E, Kydyrmanov A, Karamendin K, et al (2013) Assessment of Caspian Seal By-Catch in an Illegal Fishery Using an Interview-Based Approach. PLOS ONE 8:e67074 10.1371/journal.pone.0067074 23840590 PMC3694144

[14] Bjørge A, Ølen N, Hartvedt S, Bøthun G, Bekkby T (2002) Dispersal and bycatch mortality in gray, *Halichoerus grypus*, and harbor, *Phoca vitulina*, seals tagged at the Norwegian coast. Marine Mammal Science 18:963–976 10.1111/j.1748-7692.2002.tb01085.x

[15] Harding K, Härkönen T (1999) Development in the Baltic grey seal (Halichoerus grypus) and ringed seal (Phoca hispica) populations during the 20th century. Ambio 28:619–627.

[16] Suuronen P, Siira A, Kauppinen T, Riikonen R, Lehtonen E, et al (2006) Reduction of seal-induced catch and gear damage by modification of trap-net design: design principles for a seal-safe trap-net. Fisheries Research 79:129–138 10.1016/j.fishres.2006.02.014

[17] Hemmingsson M, Fjälling A, Lunneryd SG (2008) The pontoon trap: description and function of a seal-safe trap-net. Fisheries Research 93:357–359 10.1016/j.fishres.2008.06.013

[18] HELCOM (2006) HELCOM recommendation 27-28/2. Helsinki Commission, 8 July, 2006.

